# The Role of Social Support and Perfectionist Climate in the Development of Sports Persistence

**DOI:** 10.3390/bs16020183

**Published:** 2026-01-27

**Authors:** Benedek Tibor Tóth, Regina Bódi, Bianka Bodolai, Zsófia Ónadi, Zsófia Kohut, Karolina Eszter Kovács

**Affiliations:** Institute of Psychology, Faculty of Arts, University of Debrecen, Egyetem tér 1, 4032 Debrecen, Hungary; bene.toth@gmail.com (B.T.T.); bodiregina99@gmail.com (R.B.); bianka.bodolai3@gmail.com (B.B.); onadizsofi@gmail.com (Z.Ó.); kohut.zsofia@gmail.com (Z.K.)

**Keywords:** sports persistence, social support, perfectionist climate, young athletes, gender differences

## Abstract

Sport persistence is a key factor in maintaining a sporting career and preventing dropout from sport. Among psychological and social factors, social support plays a prominent role as a protective factor that strengthens self-esteem and reduces the risk of burnout, as does the perfectionist climate, which is motivating in a supportive environment but places psychological burdens on athletes in its maladaptive forms. The research aimed to explore the extent to which the sport persistence of young athletes can be explained by different sources of social support (parental, teacher, peer) and the dimensions of the perfectionist climate (expectations, criticism, control, conditional respect, anxiety), as well as the role played by gender. We conducted a cross-sectional, quantitative study involving 1105 young people aged 14–25 who regularly participate in sports. We used validated questionnaires to measure sports persistence, social support and perfectionist climate. We used regression and mediation models to analyse the data. According to the analyses, parental and teacher support contributed to increased sport persistence, while peer support had no significant effect. Among the dimensions of the perfectionist climate, expectations were positively related to persistence, while criticism was negatively related. Gender also indirectly influenced sports persistence, with its effect partly mediated by social support and the perfectionist climate. The results indicate that parental and teacher support, along with an emphasis on healthy expectations, are vital for strengthening sports persistence. In contrast, a critical, controlling climate was associated with lower persistence among female athletes in the present sample.

## 1. Introduction

Among athletes, the study of sport persistence, social support and perfectionist climate is of paramount importance, as these factors fundamentally influence athletic performance, mental health and long-term active participation in sport. Research on sports persistence highlights the motivational and psychological factors that help athletes cope with difficulties and maintain their sporting careers (García Calvo et al., 2010). The key role of social support is evident in its protective effect against burnout and psychological exhaustion, while strengthening self-esteem and well-being (Rees & Hardy, 2000; Sullivan et al., 2022).

In this context, the perfectionist climate has a dual effect: in some cases, it can stimulate performance, but its maladaptive forms cause significant psychological burdens, increasing the risk of stress, burnout, and depression (Crowell & Madigan, 2021; Grugan et al., 2021; Minichiello et al., 2024). The combined examination of these three factors enables the development of sports psychology interventions that support athletes’ mental health, strengthen motivation, and promote balanced performance in the long term.

### 1.1. The Role and Characteristics of Sport Persistence Among Athletes

Sport persistence is a complex concept used to describe the acquisition, form, level and effectiveness of sporting activity. It is a combination of sporting performance and mental strength that determines how an athlete can overcome difficulties, learn from failures, and develop in the long term. This concept includes personality traits such as flexibility, adaptability, and positive personality traits (K. E. Kovács, 2021).

Sport persistence, i.e., long-term commitment and perseverance, is essential for a successful sporting career, as high motivation and psychological resilience are prerequisites for outstanding results. Sport persistence refers to the long-term maintenance of sport participation and the capacity to continue engagement despite challenges, setbacks, or competing demands. The concept is closely related to earlier work on sport commitment and dropout, particularly within motivational frameworks such as self-determination theory. Seminal studies by García Calvo and colleagues (García Calvo et al., 2010) highlighted sport persistence as a key outcome reflecting sustained motivation and resistance to dropout among adolescent athletes. Subsequent research has further elaborated sport persistence as a multidimensional construct shaped by psychological resources, social environments, and contextual demands across the sporting pathway (K. E. Kovács, 2021). This indicator links physical activity with mental commitment and also indicates the likelihood of dropping out of sport (K. E. Kovács & Szakál, 2024).

Adolescents and young adults who are simultaneously engaged in education and organised sport represent a distinct population in which sport participation unfolds alongside academic demands. In this dual academic–sport context, continued engagement in sport depends not only on physical capacities but also on the ability to balance educational expectations with training and competition. Accordingly, the present study focuses on sport persistence among student athletes enrolled in secondary or higher education who participate in organised sport at either a recreational or competitive level. In this regard, one should mention that it is sometimes difficult to find a balance between academic performance and regular exercise without one compromising the other. Some studies have shown a positive correlation between participation in sports during university studies and academic performance (K. Kovács, 2015; Pinto-Escalona et al., 2022; Sun et al., 2025), while others have found a negative correlation (De Bosscher et al., 2021; K. E. Kovács, 2020; Pinto-Escalona et al., 2022).

Adolescents and young adults who are simultaneously engaged in formal education and organised sport constitute a distinct population in which sport persistence unfolds under dual academic and athletic demands. For student athletes, continued engagement in sport requires not only physical capacity and motivation, but also the ability to balance training, competition, and educational expectations (K. E. Kovács, 2020; Tóth et al., 2024). This dual-career context places particular importance on social support from parents and teachers, as well as on the evaluative climate surrounding performance. Accordingly, the present study focuses on sport persistence among secondary and higher education students who participate in sport at recreational or competitive levels, rather than on elite or professional athletes as a separate category.

Research suggests that sport persistence in this population is shaped by a combination of social and psychological factors (Consoni, 2025; K. E. Kovács & Szakál, 2024; Lane, 2024). Among these, social support from parents, teachers, and peers plays a central role in sustaining motivation and commitment, while the perceived perfectionist climate reflects evaluative pressures and expectations that may either support or undermine long-term engagement. In addition, gender-related differences in socialisation, expectations, and sport experiences may influence how these contextual factors are perceived and how they relate to persistence. For these reasons, the present study examines social support, perfectionist climate, and gender as key correlates of sport persistence in student athletes.

Although the present study focuses on social support, perfectionist climate, and gender, sport persistence is also influenced by a wide range of additional factors, including individual motivation, goal orientations, coach–athlete relationships, injury experiences, training demands, and structural characteristics of sport systems, which were beyond the scope of the current investigation.

### 1.2. The Role and Characteristics of Social Support Among Athletes

Social relationships are crucial for everyday functioning, as they ensure that individuals feel loved, respected and supported, and provide opportunities for communication. This phenomenon is referred to as social support (Cobb, 1976). Adequate social support can strengthen self-esteem by helping individuals feel that their behaviour meets social expectations and providing them with psychological stability (Cai & Lian, 2022; Liu et al., 2021; Schoofs et al., 2022).

Social support is a multidimensional construct encompassing assistance and encouragement provided by different social agents. In the context of youth and young adult sport, parental support has been consistently linked to continued participation by facilitating access to resources, emotional encouragement, and value alignment, even during later adolescence (Ntalachani et al., 2025). At the same time, peer support and friendships within sport become increasingly important during adolescence and young adulthood, as shared experiences, belonging, and mutual motivation contribute to sustained engagement (Zou et al., 2023). In educational sport contexts, teachers and coaches may also represent significant sources of support by shaping expectations, feedback, and motivational climates (Cox et al., 2021). Accordingly, the present study differentiates between multiple sources of social support when examining their associations with sport persistence. The support of the immediate social environment, including teammates, coaches, family members, and friends, plays a decisive role in athletes’ performance and psychological well-being. This factor is particularly critical during periods when athletes are unable to perform at their usual level, as positive reinforcement is key at such times (Beenen et al., 2025; Eather et al., 2023). In contrast, negative feedback can have an adverse psychological effect and further deteriorate performance (Nagy, 2018).

In a study of Korean university students, Li et al. (2024) compared levels of social support among recreational and competitive athletes. The results showed that both groups received significant social support, but that the effect was more pronounced among competitive athletes. This finding is consistent with previous studies (DeFreese & Smith, 2014; García Calvo et al., 2010; Rhind et al., 2011).

Bon and Doupona (2021) examined the level of social support after injury among female handball players. For active players, family was the most important source of support, followed by physiotherapists and teammates, while support from coaches was the least significant. The influence of the strength and conditioning coach was more substantial than that of the head coach. Retired athletes reported similar experiences, emphasising that social support accelerated recovery (Bon & Doupona, 2021; Kraemer et al., 2009).

### 1.3. The Role and Characteristics of the Perfectionist Climate Among Athletes

The perfectionist climate, i.e., an environment in which environmental factors and professional expectations focus on achieving perfection, can have a dual effect: on the one hand, it can motivate the athlete (interpreted as a challenge), but on the other hand, it can lead to increased anxiety, burnout or decreased performance (Meng et al., 2024). This atmosphere is particularly characteristic of competitive sports, where unrealistically high expectations are often placed on athletes (Grugan et al., 2021).

Its main characteristics include an emphasis on the negative consequences of mistakes, a lack of psychological security, a tendency to condition attention, and a compulsion to conform. The effects of this on athletes can be manifold: increased stress and anxiety, reduced psychological safety, maladaptive coping strategies, and impaired performance (Crowell & Madigan, 2021; Grugan et al., 2021; Minichiello et al., 2024).

The effects of a perfectionist climate are generally similar for both sexes: increased anxiety, fear of mistakes, and a higher risk of burnout. However, in aesthetic sports and dance, female athletes are more likely to face body-image-related expectations (Grugan et al., 2021). In terms of athletic level, a perfectionist climate is harmful at all levels: in a recreational environment, it reduces motivation, while at the elite level, it can lead to burnout and frustration (Crowell & Madigan, 2021). In terms of sports types, sanctions and expectations from coaches tend to dominate in team sports, while anxiety arising from full responsibility tends to dominate in individual sports. In aesthetic sports, pressure related to body ideals poses the highest risk (Grugan et al., 2021).

This research aims to explore the extent to which athletes’ persistence in sporting activities (sport persistence) can be explained by various psychological and environmental factors, with particular regard to social support (parental, teacher, peer support), the perfectionist climate (expectations, criticism, control, conditional respect, anxiety), and gender roles.

From a socio-ecological and motivational perspective, sport persistence is not determined by isolated individual characteristics but emerges from the interaction between social environments and athletes’ psychological interpretations of these environments. Social support from parents, teachers, and significant adults has been consistently linked to higher motivation, lower burnout, and sustained sport participation, partly by shaping the motivational climate in which athletes operate. Supportive relationships tend to foster adaptive forms of striving and high but attainable expectations, whereas low support or controlling interactions are associated with maladaptive perfectionism, excessive criticism, and anxiety (Crowell & Madigan, 2021; García Calvo et al., 2010; Teixeira et al., 2024).

Gender differences in sport persistence are likewise embedded in socialisation processes and differential expectations placed on male and female athletes. Prior research suggests that boys and girls experience and interpret social support and performance-related expectations differently, which may indirectly influence persistence through the perceived perfectionist climate (Grugan et al., 2021; Hu et al., 2023). Accordingly, it is theoretically plausible that social support and perfectionist climate function not only as direct predictors of sport persistence but also as mediating mechanisms through which gender-related differences and social contexts exert their effects.

Based on this, we have formulated the following hypotheses in our research:

**H1.**  *Gender has a significant influence on the level of sport persistence, and this effect is partly mediated by perceived social support, reflecting gender-specific socialisation patterns in sport.*

**H2.**  *Gender has a significant influence on the level of sport persistence, which is partly mediated by the perfectionist climate.*

**H3.**  *Perfectionist climate mediates the role of social support in the development of sport persistence.*

## 2. Methodology

### 2.1. Procedure

Data collection was conducted between 8 January 2024 to 20 June 2024 following approval from the United Ethical Review Committee for Research in Psychology (protocol code 2023-112, date of approval: 29 September 2023). Secondary school and higher education institutions were contacted directly, and participants were also recruited through sport-related networks using a snowball sampling approach. Data were collected using an online self-administered questionnaire, where participants completed a battery of self-report questionnaires assessing sport persistence, perceived social support, and perceived perfectionist climate, along with basic sociodemographic questions. Participation was voluntary and anonymous. Participation was voluntary and anonymous. Prior to data collection, all participants were informed about the purpose of the study, the voluntary nature of participation, and data confidentiality. Electronic informed consent was obtained from all participants aged 18 years or older. For participants under the age of 18, electronic informed consent was obtained from a parent or legal guardian, and adolescents provided their own assent before participation. No identifying personal data were collected. The completion of the full questionnaire package required approximately 15–20 min.

### 2.2. Sample

During the quantitative study, we collected data nationwide in Hungary, targeting young people aged 14–25 who exercise regularly at least 3 times a week and are pursuing secondary or higher education. A total of 1209 responses were received during data collection, but after data cleaning, 1105 individuals’ data proved analysable. The data collection period lasted from 8 January 2024 to 20 June 2024.

This study was designed to collect data at the national level from young people aged 14 to 25 who engage in regular sport participation. The sample was limited to secondary and higher education students in order to examine sport persistence within a dual academic–sport context, where educational demands, institutional expectations, and social support from parents and teachers are particularly relevant. The study did not aim to examine professional athletes as a distinct group. For the secondary school cohort, the primary aim was to recruit students from grades 9–13 attending sports-oriented secondary schools, as public education–type sports schools offer an appropriate context for examining both competitive and recreational athletes. These institutions pursue a dual mission by supporting athletic development alongside academic progress, thereby providing structured opportunities for adolescents involved in higher-level sport. At the same time, earlier studies indicate that a substantial share of students in sports schools participate in sport at a recreational level rather than competitively (K. E. Kovács, 2020). Moreover, such schools typically operate multiple class profiles beyond sport-specific tracks, which makes them suitable environments for identifying recreational athletes who can serve as a comparison group.

At the level of higher education, this institutional pathway could not be continued, as only the Hungarian University of Sports Science currently fulfils the criteria of a public education–type sports school in Hungary. Including a single university would not have enabled a sufficiently comprehensive analysis; therefore, competitive student athletes in higher education were planned to be recruited through university sports clubs. During the planning phase, efforts were made to achieve a sample balanced in terms of gender, settlement type, and school type. However, exclusive reliance on public education–type sports schools proved unfeasible due to refusals from several institutions to participate in the study. Consequently, the originally intended two-stage stratified sampling procedure could not be fully implemented, and snowball sampling was applied alongside direct institutional recruitment. In sum, participation was maximised through a combination of institutional outreach and snowball techniques, while recognising that the use of non-probability sampling may have introduced a degree of selection bias. Although the final sample cannot be considered fully representative of the national population of young athletes, the distribution of gender showed acceptable variability. Nevertheless, the findings should be interpreted with caution, as the use of non-probability sampling limits the generalisability of the results.

The sample comprised secondary school and university students, so the presentation of the results primarily follows the proportions of these groups (Table 1). Among secondary school students, grades 9–11 are represented in roughly equal proportions, while grades 12–13 were less frequent in the sample. (In the Hungarian education system, secondary education typically spans grades 9–12, with an additional 13th grade in some programmes. To avoid ambiguity, grade-level terminology follows the Hungarian education system, which may differ from secondary school classifications used in other countries.) In the total sample, 43.3% are secondary school students and 56.7% are higher education students. Among university students, those in the first three years (BA and BSc level) are in the majority, while the proportion of students at higher levels (MA and MSc) is significantly lower (Table 1).

### 2.3. Instruments

Validated instruments were used in this research. Accordingly, the questionnaire comprised the following sections: a sociodemographic block, a block of sports- and health-specific questions, and psychological measurement tools (sports persistence, social support, perfectionist climate). All instruments applied in the present study have previously been used in adolescent and young adult populations, including sport-related contexts. In the present study, all scales demonstrated satisfactory to excellent internal consistency, supporting their applicability in a sample of young athletes aged 14–25. Although full population-specific validation (e.g., confirmatory factor analysis by age group) was beyond the scope of this study, the use of established instruments with strong theoretical grounding and adequate reliability supports the validity of the findings (for the whole sample, see Section 2.3.1, Section 2.3.2, Section 2.3.3, Section 2.3.4 and Section 2.3.5; for the secondary and tertiary-level subsamples, see Table A1).

#### 2.3.1. Sociodemographic Questionnaire

At the beginning of the questionnaire, various sociodemographic characteristics were recorded, including gender, age, current level of education (secondary or higher) and grade, type of residence, parents’ educational attainment and occupational status, changes in family structure, number of siblings, self-assessed religiosity, and objective financial situation.

#### 2.3.2. Sports and Health-Specific Questions

After recording the sociodemographic data, sports-specific information was collected. This included the participants’ frequency of exercise (several times a day, daily, several times a week, once a week, monthly, less frequently), club membership (yes/no), time spent on weekly club and individual training (hours), type of sport (individual or team) and level of participation (international competitions, national championships, county championships, local city cups, does not compete).

#### 2.3.3. Sport Persistence Questionnaire

The Sport Persistence Questionnaire (K. E. Kovács & Csukonyi, 2025) is a 13-item measure of sport persistence that uses a single factor. The statements are to be rated on a 5-point Likert scale. Its reliability, as measured in the original study, is Cronbach’s α = 0.943. The minimum score on the composite index is 14 points, and the maximum score is 65 points. A higher score indicates higher persistence. The reliability in the current study is Cronbach’s α = 0.936.

#### 2.3.4. Short Scale of Youth’s Social Support

The questionnaire (K. E. Kovács & Mercz-Madarassy, 2022; Pluta et al., 2020) consists of 18 items and measures perceived support from parents (5 items), peers (8 items), and teachers (5 items) on a 5-point Likert scale (1 = strongly disagree, 5 = strongly agree). The maximum score on the parent and teacher subscales is 25, while the maximum score on the peer subscale is 40. The questionnaire is widely used internationally to assess social support among young people (Pluta et al., 2020) and is also available in Hungarian (original reliability: parental support: Cronbach’s α = 0.84; peer support: Cronbach’s α = 0.86; teacher support: Cronbach’s α = 0.84). In our study, the reliability of the questionnaire proved to be adequate (parental support: Cronbach’s α = 0.818; peer support: Cronbach’s α = 0.825; teacher support: Cronbach’s α = 0.835; total questionnaire: Cronbach’s α = 0.837).

#### 2.3.5. Perfectionistic Climate Questionnaire

The tool for measuring perfectionistic climate (Grugan et al., 2021) allows respondents to rate statements on a 5-point Likert scale (1 = strongly disagree, 5 = strongly agree). The questionnaire consists of 20 items and measures different aspects of climate through five subscales: expectations (McDonald’s omega = 0.88), criticism (McDonald’s omega = 0.74), control (McDonald’s omega = 0.74), conditional regard (McDonald’s omega = 0.88) and anxiousness (McDonald’s omega = 0.74). In our current study, we found the following reliability was detected: expectations (McDonald’s omega = 0.89), criticism (McDonald’s omega = 0.90), control (McDonald’s omega = 0.92), conditional regard (McDonald’s omega = 0.80) and anxiousness (McDonald’s omega = 0.91).

### 2.4. Statistical Analysis

Data analyses were conducted using Jamovi 2.3.28 statistical software. First, descriptive statistics (means, standard deviations, frequencies) were calculated to characterise the sample and the main study variables. Group differences across educational level and sport participation characteristics were examined using chi-square tests for categorical variables and analysis of variance (ANOVA) appropriate. To test the study hypotheses, a series of regression-based mediation analyses were conducted using bootstrapped confidence intervals to analyse the psychological and sociodemographic variables underlying sports persistence. The research aimed to explore the extent to which athletes’ persistence in sports can be explained by different dimensions of perceived success and prospects, as well as their interactions, with particular regard to gender, age, place of residence, changes in family structure and school status.

Although the sample comprised secondary school and higher education students, the primary analyses were conducted on the combined sample. This approach was chosen to maximise statistical power and to examine general patterns of association among social support, perfectionist climate, gender, and sport persistence across a broad developmental range of young athletes. Educational level was therefore treated as a descriptive characteristic rather than as a grouping variable in the mediation models. Prior to analysis, distributional assumptions were examined using the Shapiro–Wilk test. As the mediation analyses relied on bootstrapped confidence intervals, strict normality was not required.

## 3. Results

### 3.1. Characteristics of the Sample

The gender composition of the sample is 52.3% female, 46.9% male, and 0.8% did not specify their gender. This ratio roughly corresponds to the distribution characteristic of the total population. However, there are differences at the sub-sample level: males are overrepresented among secondary school students, while females are overrepresented among university students. In terms of the type of settlement where they live, the majority of participants live in county seats (37.3%), followed by respondents living in small towns (23.7%), villages (15.6%), the capital (12.2%) and large cities (10.2%), while only 0.9% live on farms. The distribution of the sub-samples also shows differences in this area: secondary school students are more likely to live in small towns and large cities. In contrast, university students are more likely to live in the capital and large cities.

In terms of mothers’ educational attainment, more than half of respondents (55.4%) indicated that their mothers had a higher education degree, while 41.1% indicated that their mothers had a secondary education degree. Only a small proportion reported that their mother or foster mother had at most a primary school education (1.8%), while 1.6% did not answer this question. A comparison of the sub-samples reveals significant differences: university students are more likely to be children of mothers with secondary education. In contrast, the secondary school sample is overrepresented by those whose mothers have at most a primary education.

The educational attainment of fathers shows a slightly different pattern. In the total sample, the majority of respondents (50.9%) indicated secondary education, while 44.8% indicated that their father or stepfather had higher education. The proportion of those with primary education remained low (2.5%), and a further 1.7% did not respond. A comparison of the sub-samples also reveals significant differences in this area: the secondary school sub-sample has a higher proportion of children of fathers with higher education, while the university student sub-sample has a higher proportion of children of fathers with secondary education.

Regarding the frequency of exercise, nearly half of the participants (49.5%) exercise several times a week. Those who exercise daily account for 18.5%, while those who exercise several times a week account for 14.8%, both of which are significant proportions. Among those who exercise less frequently, 9.3% exercise once a week and 7.9% exercise once a month. A comparison of the sub-samples reveals marked differences: daily and multiple-daily exercise are more common among secondary school students. In contrast, university students are more likely to exercise several times a week, once a week or once a month. This pattern is consistent with previous research showing that the proportion of competitive sports decreases with age (K. Kovács, 2022).

The proportion of individual athletes in the sample is higher (57.5%) than that of team athletes (42.5%). A comparison of the sub-samples also reveals significant differences: individual athletes are more prevalent in the higher education sample. In contrast, team athletes are more prevalent in the secondary school sample. The proportions of club and non-club athletes are almost equal (49.5% vs. 50.5%). However, there are differences within the sub-samples: club athletes are overrepresented among secondary school students, while non-club athletes are overrepresented among university students.

In the present study, competitive athletes were operationally defined as participants who reported regular participation in organised sport competitions (e.g., league, regional, national, or international level). Participants who reported engaging in organised sport or physical activity without regular competitive participation were classified as recreational athletes. In this regard, the largest proportion are recreational athletes (51.8%), while 48.2% are competitive athletes. Of the competitive athletes, 28.2% regularly participate in national championships or cups, 9.2% in county championships, 5.7% in international competitions, and 5.1% in local or city competitions. A comparison of the sub-samples reveals significant differences: competitive athletes, especially those active at the national, international and county levels, are overrepresented in the secondary school sample, while hobby and recreational athletes dominate among university students.

Table 2 presents gender-based descriptive statistics for sport persistence, perceived social support, and dimensions of the perfectionist climate. Male participants reported higher mean levels of sport persistence than female participants. With regard to social support, no gender differences were observed in peer support, whereas females reported significantly higher teacher support and males reported higher parental support. Across perfectionist climate dimensions, males showed higher mean scores for expectations, criticism, and control, while no significant gender differences were found for conditional regard. Female participants reported significantly higher levels of anxiety than males. Overall, these results indicate systematic but modest gender differences in both social support and perceived perfectionist climate variables.

### 3.2. The Role of Gender in the Development of Sports Persistence Through Social Support

**Figure 1:**
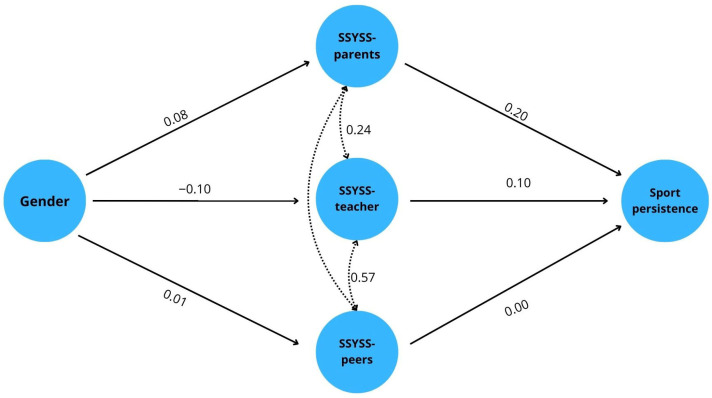
Results of the mediation analysis in relation to gender, social support and sports persistence.

All analyses presented below were conducted on the combined sample of secondary and higher education students, unless otherwise specified. The mediation analysis revealed that gender directly predicts sports persistence, with higher levels of persistence observed among males (B = −3.44; SE = 0.71; 95% CI [−4.83; −2.06]; β = −0.146; z = −4.88; *p* < 0.001). Controlling for mediators, the direct effect B remains negative and significant (B = −3.74; SE = 0.69; 95% CI [−5.09; −2.38]; β ≈ −0.159; *p* < 0.001), which is consistent with the group averages (Sports persistence: women = 51.59; men = 48.15) (see Figure 1 and Table 3).

Regarding the indirect effect, two paths with opposite signs emerge. On the one hand, *parental support* mediates non-B in a positive direction: men report higher parental support (parental support: women = 18.42; men = 19.62), and parental support is positively associated with sport persistence (indirect B: ab* = 0.52; SE = 0.17; 95% CI [0.21; 0.88]; β = 0.022; z = 3.05; *p* = 0.002; b-path: B = 0.431; SE = 0.069; β = 0.212; *p* < 0.001). On the other hand, the *teacher support* path shows negative mediation: women perceive higher teacher support (teacher support: women = 15.39; men = 14.21), while teacher support is also positively related to sports persistence, thus men’s lower teacher support pulls persistence down (B: a-path = −0.22; SE = 0.11; 95% CI [−0.48; −0.04]; β = −0.010; z = −2.00; *p* = 0.046; b-path: B = 0.191; SE = 0.068; β = 0.100; *p* = 0.005). In the case of *peer support*, no significant mediation can be demonstrated (ab* = 0.00; SE = 0.03; 95% CI [−0.07; 0.08]; β ≈ 0.000; z = 0.06; *p* = 0.951), and path b is also insignificant (B = 0.006; SE = 0.052; β ≈ 0.005; *p* = 0.907). The combined indirect effect of the three mediators is small and insignificant (B = 0.30; SE = 0.22; 95% CI [−0.12; 0.73]; β = 0.013; z = 1.35; *p* = 0.176), suggesting that positive parental and negative teacher mediation partially cancel each other out. Overall, therefore, the effect of gender on sport persistence is predominantly direct. At the same time, through the components of social support, the parental branch slightly strengthens, and the teacher branch weakens this relationship.

Although several paths reached statistical significance, the corresponding standardized coefficients indicated small effect sizes, suggesting modest associations rather than strong predictive relationships.

### 3.3. The Role of Gender in the Development of Sport Persistence Through the Perfectionist Climate

**Figure 2:**
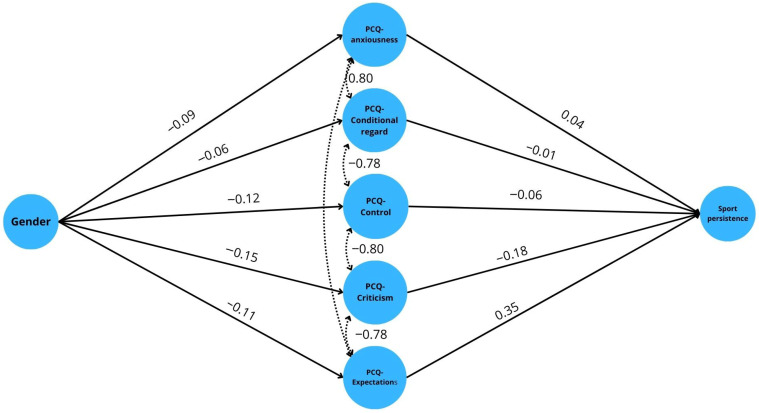
Results of the mediation analysis regarding gender, perfectionist climate and sports persistence.

Based on the results, gender directly influences sport persistence (β = –0.131, z = –4.429, *p* < 0.001), and this relationship also manifests indirectly through specific dimensions of the Perfectionist Climate (PCQ). Mediation analysis showed that expectations and, in particular, criticism play a significant mediating role (see Figure 2 and Table 4).

Gender did not significantly predict perceptions of higher expectations (β = –0.111, z = –3.724, *p* < 0.001), whereas expectations were strongly positively related to the level of sport persistence (β = 0.347, z = 6.076, *p* < 0.001). The indirect effect was significant (B = –0.883, SE = 0.278, 95% CI [–1.427; –0.338], β = –0.039, z = –3.175, *p* = 0.001), suggesting that gender also contributes to the development of persistence levels through expectations.

Significant correlations were also found for the criticism dimension. Gender did not negatively influence the perception of criticism (β = –0.146, z = –4.910, *p* < 0.001), and criticism was negatively related to sports persistence (β = –0.179, z = –2.841, *p* = 0.004). The indirect effect was also significant in this case (B = 0.600, SE = 0.244, 95% CI [0.122; 1.077], β = 0.026, z = 2.459, *p* = 0.014), suggesting that higher perceived criticism reduces persistence levels, especially among women.

However, we did not find a convincing mediating effect for other dimensions. Although control was a non-significant predictor (β = –0.119, z = –3.999, *p* < 0.001), the relationship between control and sports persistence was also non-significant (β = –0.057, z = –1.061, *p* = 0.289), so no indirect effect was detectable. In the case of conditional respect, the effect of gender was only a weak, borderline predictor (β = –0.057, z = –1.888, *p* = 0.059), which showed no relationship with sport persistence (β = –0.009, z = –0.156, *p* = 0.876), so its mediating role was not confirmed here either. Similarly, in the anxiety dimension, gender significantly predicted higher anxiety values (β = –0.086, z = –2.856, *p* = 0.004), with female participants reporting higher anxiety scores than male participants. However, the relationship between anxiety and sports persistence was not significant (β = 0.039, z = 0.649, *p* = 0.516), so the indirect effect was also not significant.

Overall, the total effect (direct + indirect) remained significant (B = –3.178, SE = 0.681, 95% CI [–4.514; –1.843], β = –0.139, z = –4.664, *p* < 0.001). This means that the relationship between gender and sport persistence can be partly explained by certain aspects of the perfectionist climate, particularly expectations and criticism. At the same time, no mediating effect was found for other dimensions (control, conditional respect, anxiety).

**Figure 3:**
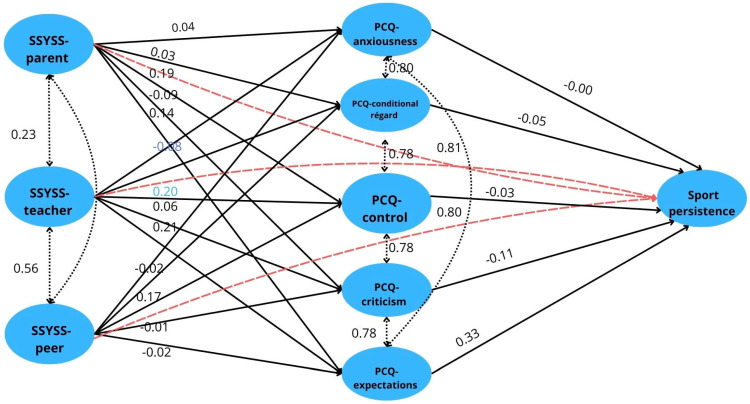
Results of the mediation analysis in relation to social support, perfectionist climate and sport persistence.

The effect of social support on sport persistence can be examined across several sources. Based on direct effects, both teacher support (B = 0.200, SE = 0.068, β = 0.104, z = 2.96, *p* = 0.003, 95% CI [0.068; 0.333]) and parental support (B = 0.324, SE = 0.070, β = 0.160, z = 4.65, *p* < 0.001, 95% CI [0.188; 0.461]) significantly increase sports persistence. However, no significant direct relationship can be demonstrated in the case of peer support (B = 0.003, SE = 0.051, β = 0.002, z = 0.06, *p* = 0.956, 95% CI [−0.097; 0.103]) (see Figure 3 and Table 5).

Among the indirect effects, the relationship between teacher support and the “expectations” dimension of perfectionism stands out. Teacher support significantly increases the level of expectations (B = 0.147, SE = 0.031, β = 0.171, z = 4.75, *p* < 0.001, 95% CI [0.086; 0.207]), which in turn strongly predicts sports persistence (B = 0.727, SE = 0.126, β = 0.325, z = 5.77, *p* < 0.001, 95% CI [0.480; 0.973]). The mediation result is also significant (B = 0.106, SE = 0.029, β = 0.056, z = 3.67, *p* < 0.001, 95% CI [0.050; 0.163]), indicating that teacher support indirectly contributes to higher levels of sport persistence through expectations.

Teacher support is also related to other dimensions of perfectionism (criticism: B = 0.166, SE = 0.029, β = 0.208, z = 5.81, *p* < 0.001; control: B = 0.147, SE = 0.027, β = 0.198, z = 5.52, *p* < 0.001; conditional respect: B = 0.116, SE = 0.030, β = 0.141, z = 3.89, *p* < 0.001; anxiety: B = 0.157, SE = 0.030, β = 0.190, z = 5.30, *p* < 0.001); however, these do not significantly mediate sports persistence (e.g., criticism → sports persistence: B = −0.271, SE = 0.151, β = −0.113, z = −1.80, *p* = 0.072).

The indirect effects of parental support are less pronounced. Although there was a near-significant relationship in the direction of “expectations” (B = 0.058, SE = 0.032, β = 0.064, z = 1.83, *p* = 0.068), the indirect effect on sport persistence did not reach the level of significance (B = 0.042, SE = 0.024, β = 0.021, z = 1.74, *p* = 0.082). However, interestingly, parental support showed a negative correlation with the “criticism” dimension of perfectionism (B = −0.065, SE = 0.029, β = −0.077, z = −2.23, *p* = 0.026) and “control” dimensions of perfectionism (B = −0.070, SE = 0.027, β = −0.088, z = −2.54, *p* = 0.011), which may indicate that higher parental support reduces the negative perfectionist pressure experienced by athletes.

Peer support did not show a significant relationship with any of the perfectionism dimensions (e.g., expectations: B = −0.014, SE = 0.024, β = −0.025, z = −0.60, *p* = 0.549), so no indirect effect can be confirmed.

Based on the total effects, teacher support (B = 0.236, SE = 0.068, β = 0.123, z = 3.48, *p* < 0.001) and parental support (B = 0.385, SE = 0.070, β = 0.190, z = 5.54, *p* < 0.001) both contribute significantly to the level of sport persistence. In contrast, peer support does not show a detectable total effect (B = −0.004, SE = 0.052, β = −0.003, z = −0.07, *p* = 0.946).

## 4. Discussion

From a broader theoretical perspective, the present findings align with socio-ecological and motivational models of sport participation, which emphasise the role of interpersonal environments in shaping athletes’ engagement and persistence. Rather than acting as isolated influences, social support and perfectionism-related climate appear to operate as interrelated contextual factors that are associated with sustained sport involvement. This supports previous research suggesting that athletes’ motivational experiences are embedded within social expectations and evaluative processes rather than driven solely by individual traits (Crowell & Madigan, 2021; García Calvo et al., 2010; Teixeira et al., 2024).

Before interpreting the findings, it is important to note that the observed associations and indirect effects were generally small in magnitude, which is typical in research on complex psychosocial outcomes such as sport persistence. Regarding our results, H1 was partially confirmed: the effect of gender was strong, but the mediating role of social support was mixed and overall weak. One central finding of the mediation analysis is that gender shows a statistically significant association with sport persistence, and the findings suggest that targeted forms of social support may represent one potential avenue, among others, for addressing gender differences in sport persistence. In the present sample, men show lower levels of persistence on average than women which is consistent with previous research findings (K. E. Kovács, 2025), and this difference remains even when social support is taken into account (Thibault et al., 2010). The mediating pathways show a dual pattern: parental support is stronger for boys and independently increases persistence (Gao et al., 2024; Lee & Park, 2021), so the effect of gender is mediated positively on this branch. In contrast, teacher support is higher for girls and is also positively related to persistence; since boys receive less of this, this channel mediates the effect of gender in a negative direction (Pautu et al., 2025). The two mediations largely cancel each other out, so the overall indirect effect is not significant. Peer support does not play a significant mediating role. This suggests that, in addition to direct, gender-related factors, differences in perceived social support may contribute modestly to gender differences in sport persistence (Gurieva et al., 2022; Uhing et al., 2021). The findings suggest that different sources of adult support may be experienced as more salient by different groups within the sample; however, these patterns should be interpreted cautiously and not as prescriptive gender-based recommendations. For boys, teacher support may represent a potentially relevant contextual focus for future interventions (teacher-student relationships, regular feedback, coach-school cooperation), while for girls, increasing parental involvement (consistent recognition, training and competition support, encouragement at home) may be beneficial. Since peer influence has not proven to be a relevant mediator, it is better to focus programme resources on the quality of family and school/teacher support.

In addition, H2 was partially confirmed: specific dimensions of the perfectionist climate (expectations, criticism) indeed mediated the relationship between gender and sport persistence. Gender-related differences in sport persistence were reflected in certain aspects of the perceived perfectionist climate, likely shaped by socialisation processes and sport-specific expectations. Among the examined dimensions, expectations and criticism showed statistically significant, though modest, mediating association, which reflect how aspects of the perceived environment are associated with variations in sport persistence, especially the expectations of coaches and peers, as well as critical feedback, are associated with variations in the level of persistence. According to the results, the perception of higher expectations, although often perceived as pressure, was positively related to persistence. This suggests that, for athletes, the structuring and motivating power of expectations can strengthen persistence when experienced in a supportive context (Martín-Rodríguez et al., 2024; Schüler et al., 2023). In contrast, the criticism dimension had a negative effect: an increase in the critical atmosphere was associated with a decrease in sports persistence (Hu et al., 2023). It is particularly noticeable among female athletes that excessive or destructive criticism weakens long-term persistence, highlighting the importance of the quality and manner of feedback (Damián Núñez et al., 2024). Overall, gender differences observed in this study should be interpreted as context-dependent patterns shaped by social, cultural, and sport-specific factors, rather than as fixed or universal gender characteristics.

Finally, H3 was confirmed: the effect of teacher (and, to a lesser extent, parental) support was mediated by the perfectionist climate, but not across all dimensions or for all sources. The analysis suggests that teacher and parental support are among several relevant social factors associated with sport persistence. Both show statistically significant but modest direct associations with sport persistence, but teacher support also has an indirect effect: it further positively impacts persistence through the dimension of perfectionism “expectations”. This suggests that teacher reinforcement perceived by athletes is associated with higher persistence and with higher perceived expectations, which may be perceived by athletes as motivating under certain conditions. Although parental support did not show a significant indirect effect, it serves as an important protective factor by reducing the criticism and control experienced by the athlete, thereby mitigating the pressure associated with harmful perfectionism (Teixeira et al., 2024). In contrast, peer support did not significantly influence sports persistence, either directly or indirectly, suggesting that adults—especially teachers and parents—may play a much more prominent role than peers in relation to sports persistence in the current sample. Future research should build on these findings by examining whether the observed associations differ across developmental stages, sport types, or competitive levels. Longitudinal designs would be particularly valuable for testing the temporal ordering of social support, perceived perfectionist climate, and sport persistence. In addition, qualitative or mixed-method approaches could provide deeper insight into how athletes interpret expectations and support within specific sport contexts.

### Strengths and Limitations

The present study has several notable strengths. It is based on a large and heterogeneous nationwide sample of secondary and higher education student athletes, covering a broad developmental range from adolescence to young adulthood. Also, the study differentiates between multiple sources of social support and multiple dimensions of the perfectionist climate, allowing for a nuanced examination of their distinct and combined associations with sport persistence. In addition, the use of theoretically grounded mediation models provides insight into potential psychological pathways linking social contexts and persistence, thereby extending previous research that has primarily focused on direct associations.

However, the study is not without limitations. One limitation of the study is the non-representative sampling, which means the results cannot be generalised to the entire population of athletes. The sampling procedure was based on a combination of institutional recruitment and snowball sampling, which constitutes a non-probability sampling strategy. Although this approach allowed access to a large and heterogeneous group of young athletes, it inevitably increases the risk of selection bias. In particular, participants who were more engaged in sport, embedded in supportive social networks, or affiliated with sport-oriented institutions may have been more likely to take part in the study, while less connected or more marginalised athletes may be underrepresented. As a consequence, the final sample cannot be considered fully representative of the national population of young athletes in Hungary. Despite our efforts to achieve a balanced gender distribution in our sample, men were overrepresented. However, the gender distribution observed in the current sample is consistent with previous research, which indicates a higher participation rate among men in various sports activities (Eime et al., 2021). Therefore, the findings should not be interpreted as population-level estimates but rather as indicative of associations within the recruited sample.

In addition, the cross-sectional design of the study represents an important limitation, particularly with respect to the mediation analyses. Although mediation models were used to examine indirect associations among social support, perfectionist climate, and sport persistence, the present design does not allow conclusions about temporal ordering or causal relationships between these variables. Consequently, the identified indirect effects should be interpreted as statistical and theoretical associations rather than evidence of causal mechanisms. Longitudinal or experimental studies are required to test whether changes in social support precede changes in perceived perfectionist climate and, in turn, influence sport persistence over time.

Although several potentially relevant background variables (e.g., age, level of competition, type of sport, training frequency, and indicators of socioeconomic status) were collected, these factors were not included as covariates in the mediation models. As a result, the observed associations may partly reflect unmeasured differences related to sport-specific demands, training load, or social background. The mediation analyses were therefore designed to explore theoretically grounded psychological pathways rather than to provide fully adjusted estimates. Consequently, the findings should be interpreted with caution, as indirect effects may vary across age groups, sport types, or competitive levels. Future studies should apply longitudinal designs and incorporate systematic covariate control to test the robustness and generalisability of the proposed mediation mechanisms.

In addition, although differences between secondary and higher education students are theoretically relevant, subgroup-specific mediation analyses were beyond the scope of the present study and would require larger, stratified samples to ensure adequate statistical power.

Additionally, no formal robustness checks, sensitivity analyses, or alternative model specifications were conducted. Although the mediation analyses relied on bootstrapped confidence intervals and tested multiple mediators simultaneously, the stability of the observed indirect effects across alternative model structures (e.g., reversed mediation, covariate-adjusted models, or subgroup-specific analyses) was not examined. Consequently, the findings should be interpreted as exploratory and hypothesis-generating rather than as definitive evidence for specific mediation pathways. Future research should explicitly test alternative theoretical models, apply sensitivity analyses, and examine whether the proposed mechanisms remain robust when key background variables are systematically controlled.

It is important to note that the effect sizes observed in the present study were generally small. This is consistent with previous research on sport persistence and related motivational outcomes, which are known to be influenced by a wide range of individual, social, and contextual factors. Consequently, even statistically significant effects are unlikely to account for a large proportion of variance in persistence on their own. The findings should therefore be interpreted as identifying meaningful but modest associations that contribute to a broader, multifactorial understanding of sport persistence rather than as evidence of strong or dominant effects.

## 5. Conclusions

The present study examined the associations between social support, perceived perfectionist climate, gender, and sport persistence among secondary and higher education student athletes. The findings indicate that sport persistence is modestly but consistently associated with multiple sources of social support and with dimensions of the perceived perfectionist climate, highlighting the importance of social and evaluative environments in sustained sport engagement. The results suggest that parental and teacher support, as well as supportive and non-critical performance climates, are relevant contextual factors associated with sport persistence, while gender-related differences appear to be embedded in broader socialisation and expectation processes rather than reflecting inherent characteristics.

The findings of the present study have several practical implications for educational and sport settings. The results highlight the importance of parental and teacher support in fostering sport persistence among young athletes, suggesting that interventions should prioritise strengthening supportive adult–athlete relationships. In particular, teachers and coaches may play a key role by providing clear but attainable expectations and constructive feedback, while avoiding excessive criticism and controlling practices. Furthermore, the results underline the need for awareness of gender-sensitive dynamics in sport environments. Although the observed effects are modest, they point to actionable areas where educational institutions, coaches, and sport psychologists can support sustained engagement in sport.

## Figures and Tables

**Table 1:** Characteristics of the sample along sociodemographic data (N = 1105).

		Secondary Level			Tertiary Level			*p*
		N	Row%	Adj Res	N	Row %	Adj Res	
Gender	Male	282	54.4%	7.0	236	45.6%	−7.0	<0.001
	Female	194	33.6%	−6.8	383	66.4%	6.8	
	I do not wish to answer	2	22.2%	−1.3	7	77.8%	1.3	
	Total	478	43.3%		626	56.7%		
Type of settlement	Capital	47	34.8%	−2.1	88	65.2%	2.1	<0.001
	County seat	153	37.1%	−3.2	259	62.9%	3.2	
	Big city	61	54.0%	2.4	52	46.0%	−2.4	
	Small town	145	55.3%	4.5	117	44.7%	−4.5	
	Village	67	39.0%	−1.3	105	61.0%	1.3	
	Farm	5	50.0%	0.4	5	50.0%	−0.4	
	Total	478	43.3%		626	56.7%		
Mother’s education	primary	13	65.0%	2.0	7	35.0%	−2.0	<0.001
	secondary	172	37.9%	−3.0	282	62.1%	3.0	
	tertiary	275	44.9%	1.2	337	55.1%	−1.2	
	does not know	18	100.0%	4.9	0	0.0%	−4.9	
	Total	478	43.3%		626	56.7%		
Father’s education	primary	13	46.4%	0.3	15	53.6%	−0.3	<0.001
	secondary	215	38.3%	−3.4	347	61.7%	3.4	
	tertiary	231	46.7%	2.0	264	53.3%	−2.0	
	does not know	19	100.0%	5.0	0	0.0%	−5.0	
	Total	478	43.3%		626	56.7%		

**Table 2:** The characteristics of the psychological variables measured by gender.

Gender		Sport Persistence	SSYSS- Peer	SSYSS- Teacher	SSYSS- Parent	PCQ- Expectations	PCQ- Criticism	PCQ- Control	PCQ- Conditional Regard	PCQ- Anxiousness
Mean	female	48.1	27.5	14.2	19.6	10.0	8.3	7.2	9.2	9.5
	male	51.6	27.2	15.4	18.4	11.2	9.7	8.4	9.7	10.4
Median	female	50.0	29.0	15.0	22.0	9.0	7.0	5.0	8.0	9.0
	male	54.0	28.5	16.0	20.0	11.0	9.0	7.0	9.0	10.0
Standard deviation	female	12.1	9.3	5.9	5.8	5.2	4.8	4.4	5.1	5.0
	male	11.1	8.9	6.4	5.8	5.3	5.0	4.7	5.1	5.2
Minimum	female	13.0	8.0	5.0	5.0	4.0	4.0	4.0	4.0	4.0
	male	13.0	8.0	5.0	5.0	4.0	4.0	3.0	3.0	4.0
Maximum	female	65.0	40.0	25.0	25.0	20.0	20.0	20.0	20.0	20.0
	male	65.0	40.0	25.0	25.0	20.0	20.0	20.0	20.0	20.0
Significance	*p*	<0.001	0.393	0.001	<0.001	<0.001	<0.001	<0.001	0.084	0.005

**Table 3:** Results of the mediation analysis for gender, social support and sport persistence.

				95% C.I. (a)				
Type	Effect	Estimate	SE	Lower	Upper	β	z	*p*
Indirect	Gender ⇒ SSYSS-contemporary ⇒ Sports persistence	3.50 × 10^−4^	0.00586	−0.0111	0.011	1.53 × 10^−5^	0.0598	0.952
	Gender ⇒ SSYSS teacher ⇒ Sports persistence	−0.232	0.10651	−0.441	−0.0242	−0.0102	−2.1874	0.029
	Gender ⇒ SSYSS parent ⇒ Sports persistence	0.384	0.1537	0.082	0.6856	0.0168	2.4987	0.012
Component	Gender ⇒ SSYSS-contemporary	0.10859	0.53197	−0.9340	1.1512	0.00614	0.2041	0.838
	SSYSS-contemporary ⇒ Sport persistence	0.00323	0.05158	−0.097	0.1043	0.0025	0.0625	0.950
	Gender ⇒ SSYSS teacher	−1.18631	0.35696	−1.8860	−0.4867	−0.09952	−3.3233	<0.001
	SSYSS teacher ⇒ Sports persistence	0.19639	0.06759	0.0639	0.3289	0.10244	2.9054	0.004
	Gender ⇒ SSYSS parent	0.92807	0.33764	0.2663	1.5898	0.08244	2.7487	0.006
	SSYSS parent ⇒ Sports persistence	0.414	0.06904	0.2787	0.5493	0.20395	5.9969	<0.001
Direct	Gender ⇒ Sports persistence	−3.32969	0.66647	−4.6359	−2.0234	−0.14571	−4.9960	<0.001
Total	Gender ⇒ Sports persistence	−3.17809	0.68136	−4.5135	−1.8426	−0.13908	−4.6643	<0.001

**Table 4:** Results of the mediation analysis for gender, perfectionist climate and sport persistence.

				95% C.I. (a)				
Type	Effect	Estimate	SE	Lower	Upper	β	z	*p*
Indirect	Gender ⇒ PCQ expectations ⇒ Sports persistence	−0.8827	0.2780	−1.427	−0.3378	−0.03863	−3.175	0.001
	Gender ⇒ PCQ criticism ⇒ Sports persistence	0.5995	0.243	0.122	1.0773	0.02623	2.459	0.014
	Gender ⇒ PCQ control ⇒ Sports persistence	0.154	0.1510	−0.141	0.4507	0.0067	1.025	0.305
	Gender ⇒ PCQ conditional respect ⇒ Sports persistence	0.012	0.078	−0.141	0.1654	5.33 × 10^−4^	0.156	0.876
	Gender ⇒ PCQ anxiety ⇒ Sports persistence	−0.0769	0.1214	−0.315	0.161	−0.00337	−0.633	0.527
Component	Gender ⇒ PCQ expectations	−1.1394	0.305	−1.739	−0.5398	−0.11139	−3.724	<0.001
	PCQ expectations ⇒ Sports persistence	0.774	0.1275	0.525	1.0246	0.34677	6.076	<0.001
	Gender ⇒ PCQ criticism	−1.3894	0.2830	−1.944	−0.8347	−0.14618	−4.910	<0.001
	PCQ criticism ⇒ Sports persistence	−0.4315	0.1519	−0.729	−0.1338	−0.17946	−2.841	0.004
	Gender ⇒ PCQ control	−1.0613	0.2654	−1.582	−0.5411	−0.11949	−3.999	<0.001
	PCQ control ⇒ Persistence in sport	−0.1458	0.1375	−0.415	0.1236	−0.05669	−1.061	0.289
	Gender ⇒ PCQ conditional respect	−0.557	0.295	−1.135	0.0212	−0.05674	−1.888	0.059
	PCQ conditional respect ⇒ Sport persistence	−0.0218	0.139	−0.296	0.252	−0.00939	−0.156	0.876
	Gender ⇒ PCQ anxiety	−0.8446	0.295	−1.424	−0.2649	−0.08564	−2.856	0.004
	PCQ anxiety ⇒ Persistence in sport	0.091	0.1402	−0.184	0.365	0.0393	0.649	0.516
Direct	Gender ⇒ Sports persistence	−2.9850	0.6739	−4.306	−1.6641	−0.13063	−4.429	<0.001
Total	Gender ⇒ Sports persistence	−3.1781	0.6814	−4.514	−1.8426	−0.13908	−4.664	<0.001

**Table 5:** Results of the mediation analysis for social support, perfectionist climate, and sport persistence.

				95% C.I. (a)				
Type	Effect	Estimate	SE	Lower	Upper	β	z	*p*
Indirect	SSYSS-contemporary ⇒ PCQ expectations ⇒ Sports persistence	−0.01031	0.01731	−0.04423	0.0236	−0.00797	−0.5956	0.551
	SSYSS-contemporary ⇒ PCQ-criticism ⇒ Sport persistence	0.0019	0.00604	−0.00995	0.01374	0.00147	0.3138	0.754
	SSYSS-contemporary ⇒ PCQ-control ⇒ Sports persistence	5.77 × 10^−4^	0.00184	−0.00304	0.00419	4.46 × 10^−4^	0.3128	0.754
	SSYSS-contemporary ⇒ PCQ-conditional respect ⇒ Sport persistence	0.00135	0.00306	−0.00465	0.00735	0.00104	0.4405	0.660
	SSYSS-contemporary ⇒ PCQ anxiety ⇒ Sports persistence	1.72 × 10^−4^	0.00214	−0.00403	0.00437	1.33 × 10^−4^	0.0802	0.936
	SSYSS teacher ⇒ PCQ expectations ⇒ Sports persistence	0.10646	0.02904	0.0495	0.16337	0.05553	3.6663	<0.001
	SSYSS teacher ⇒ PCQ criticism ⇒ Sports persistence	−0.04501	0.02616	−0.09627	0.00625	−0.02348	−1.7208	0.085
	SSYSS teacher ⇒ PCQ control ⇒ Sports persistence	−0.01074	0.02017	−0.05028	0.02879	−0.00560	−0.5326	0.594
	SSYSS teacher ⇒ PCQ conditional respect ⇒ Sports persistence	−0.01307	0.01636	−0.04513	0.0189	−0.00682	−0.7990	0.424
	SSYSS teacher ⇒ PCQ anxiety ⇒ Sports persistence	−0.00176	0.02181	−0.04452	0.04099	−9.19 × 10^−4^	−0.0808	0.936
	SSYSS parent ⇒ PCQ expectations ⇒ Sports persistence	0.04197	0.02409	−0.00525	0.089	0.02068	1.7420	0.082
	SSYSS parent ⇒ PCQ criticism ⇒ Sports persistence	0.01772	0.01264	−0.00705	0.0425	0.00873	1.4019	0.161
	SSYSS parent ⇒ PCQ control ⇒ Sports persistence	0.005	0.0097	−0.01392	0.02408	0.0025	0.5237	0.601
	SSYSS parent ⇒ PCQ conditional respect ⇒ Sports persistence	−0.00330	0.00531	−0.01371	0.00711	−0.00163	−0.6219	0.534
	SSYSS parent ⇒ PCQ anxiety ⇒ Sports persistence	−3.77 × 10^−4^	0.00469	−0.00956	0.00881	−1.86 × 10^−4^	−0.0806	0.936
Component	SSYSS-contemporary ⇒ PCQ expectations	−0.01419	0.02369	−0.06063	0.03225	−0.02452	−0.5988	0.549
	PCQ expectations ⇒ Sports persistence	0.7265	0.12602	0.4795	0.97349	0.3252	5.7651	<0.001
	SSYSS-contemporary ⇒ PCQ-criticism	−0.00699	0.02193	−0.04997	0.03599	−0.01300	−0.3187	0.750
	PCQ criticism ⇒ Sports persistence	−0.27137	0.15062	−0.56657	0.02384	−0.11287	−1.8017	0.072
	SSYSS-contemporary ⇒ PCQ-control	−0.00791	0.0205	−0.04815	0.0323	−0.01575	−0.3855	0.700
	PCQ control ⇒ Sports persistence	−0.0729	0.1362	−0.33989	0.19409	−0.02834	−0.5351	0.593
	SSYSS-contemporary ⇒ PCQ-conditional respect	−0.01198	0.02289	−0.05685	0.03289	−0.02157	−0.5232	0.601
	PCQ-conditional respect ⇒ Sport persistence	−0.11264	0.13798	−0.38308	0.1578	−0.04839	−0.8163	0.414
	SSYSS-contemporary ⇒ PCQ anxiety	−0.01535	0.02282	−0.06007	0.02937	−0.02752	−0.6729	0.501
	PCQ anxiety ⇒ Sports persistence	−0.01119	0.1385	−0.28267	0.26030	−0.00483	−0.0808	0.936
	SSYSS teacher ⇒ PCQ expectations	0.146	0.03085	0.08608	0.20699	0.17076	4.7507	<0.001
	SSYSS teacher ⇒ PCQ criticism	0.16586	0.02855	0.10991	0.22182	0.20802	5.8099	<0.001
	SSYSS teacher ⇒ PCQ control	0.14738	0.02672	0.09501	0.19976	0.19778	5.5152	<0.001
	SSYSS teacher ⇒ PCQ conditional respect	0.11603	0.0298	0.05762	0.17445	0.14088	3.8932	<0.001
	SSYSS teacher ⇒ PCQ anxiety	0.15747	0.0297	0.09926	0.21569	0.19033	5.3016	<0.001
	SSYSS parent ⇒ PCQ expectations	0.057	0.03161	−0.00419	0.11973	0.06358	1.8274	0.068
	SSYSS parent ⇒ PCQ criticism	−0.06531	0.02926	−0.12266	−0.00796	−0.07735	−2.2320	0.026
	SSYSS parent ⇒ PCQ control	−0.06965	0.02739	−0.1233	−0.01597	−0.08826	−2.5429	0.01
	SSYSS parent ⇒ PCQ conditional respect	0.0293	0.03055	−0.03054	0.0892	0.03363	0.9600	0.337
	SSYSS parent ⇒ PCQ anxiety	0.03374	0.0304	−0.02593	0.0934	0.03851	1.1082	0.268
Direct	SSYSS-contemporary ⇒ Sport persistence	0.0028	0.051	−0.09717	0.10278	0.00217	0.0550	0.956
	SSYSS teacher ⇒ Sports persistence	0.20017	0.06766	0.06755	0.33278	0.10441	2.9582	0.003
	SSYSS parent ⇒ Sports persistence	0.32434	0.06975	0.18763	0.46104	0.15978	4.6501	<0.001
Total	SSYSS-contemporary ⇒ Sports persistence	−0.00351	0.05217	−0.10576	0.09874	−0.00271	−0.0672	0.946
	SSYSS teacher ⇒ Sports persistence	0.236	0.06791	0.10293	0.36915	0.12313	3.4757	<0.001
	SSYSS parent ⇒ Persistence in sport	0.38543	0.06960	0.24900	0.52185	0.18987	5.5374	<0.001

## Data Availability

Data are available only on request due to ethical restrictions.

## Abbreviations

The following abbreviations are used in this manuscript:

SOQ	Sport Orientation Questionnaire
SSYSS	Short Scale of Youth’s Social Support

## Appendix A

**Table A1:** Reliability of the instruments along with the secondary and tertiary-level subsamples.

	Secondary School Subsample	University Subsample
SPQ	α = 0.94	α = 0.94
SSYSS—parental support	α = 0.84	α = 0.85
SSYSS—peer support	α = 0.87	α = 0.86
SSYSS—teacher support	α = 0.83	α = 0.84
PCQ—expectations	ω = 0.89	ω = 0.90
PCQ—criticism	ω = 0.91	ω = 0.89
PCQ—control	ω = 0.92	ω = 0.92
PCQ—conditional regard	ω = 0.80	ω = 0.81
PCQ—anxiousness	ω = 0.91	ω = 0.92
